# Cryoablation as a treatment option for medically inoperable NSCLC: Presentation of 2 cases

**DOI:** 10.1016/j.radcr.2025.04.123

**Published:** 2025-05-29

**Authors:** Eleni D. Eleftheriadou, Dionysia-Dimitra Samara, Maria Saroglou, Evangelos Petsatodis

**Affiliations:** a Department of Pulmonary Medicine, George Papanikolaou General Hospital, Thessaloniki 57010, Greece; b Department of Interventional Radiology, Georgios Papanikolaou General Hospital of Thessaloniki, Thessaloniki 57010, Greece

**Keywords:** Ablation, Cryoablation, Non-small cell lung cancer, Early-stage, Case report

## Abstract

In recent years, significant efforts have been made worldwide to implement lung cancer screening programs. As a result, an increasing number of patients are being diagnosed with early-stage lung cancer, where treatment options offer higher survival rates. However, approximately 25% of these patients cannot undergo surgery due to poor respiratory function because of their primary lung disease. Image-Guided thermal ablation (IGTA) techniques are recommended by the international guidelines as an option for the medically inoperable stage IA. However, due to the need for specialized centers for their application, this technique has not yet been widely adopted. The aim of this presentation is to highlight the promising results of cryoablation as a thermal ablation technique for patients with stage IA lung cancer and severely compromised lung function. This will be illustrated by 2 cases of medically inoperable early-stage non-small cell lung cancer (NSCLC) where this treatment option was successfully employed. The procedure was well tolerated and both patients were discharged from the hospital 2 days after the procedure. The follow-up included a computed tomography (CT) scan in 1, 3, 6, and 12 months and a positron emission tomography (PET) scan in 6 and 12 months. Both patients had no local recurrence of the disease for at least 1 year afterwards. These cases highlight that cryoablation, a widely approved and guideline-endorsed technique, can be safely and effectively applied in patients with significantly impaired lung function or multiple comorbidities, offering a valuable alternative when surgery is not feasible.

## Introduction

The widespread use of low-dose computed tomography in the context of lung cancer screening has been proven effective in the early diagnosis of a significant percentage of high-risk patients. Nevertheless, according to the National Cancer Database, approximately 25% of these patients are unable to undergo surgery due to their poor general condition, often exacerbated by accompanying respiratory diseases [1].

Medically inoperable patients, also called “high-risk,” are identified during preoperative evaluation based on specific cardiopulmonary criteria. Major criteria include low spirometry parameters like Forced Expiratory Volume in 1 Second (FEV1) or diffusing capacity of the lung for carbon monoxide (DLCO) ≤50%, while minor criteria include FEV1 or DLCO between 51% and 60%, age ≥75 years, pulmonary hypertension, left ventricular ejection fraction ≤40%, resting or exercise PaO₂ <55 mmHg, and pCO₂ >45 mmHg [2,3]. One major or 2 minor criteria are required to classify a patient as high-risk [2,3]. The CHEST guidelines recommend cardiopulmonary exercise testing (CPET) for all high-risk patients; however, CPET requires specialized staff and facilities and is not widely available. A simpler, nonspecific alternative is assessing if the patient can climb 2 flights of stairs without stopping. In practice, it is important to consider the patient’s overall condition and the available diagnostic tools and to conduct multidisciplinary team discussions, which have been associated with improved overall survival [3].

The treatment options for the patients that cannot undergo surgery are limited. Less invasive therapeutic strategies are recommended, like stereotactic body radiotherapy therapy (SBRT), which is the standard method of care for treating early-stage peripheral lung tumors. SBRT reduces the risk of local recurrence, achieving good primary tumor control with a 5-year survival rate of approximately 30% [4,5]. However, SBRT is associated with severe toxicity rates, up to 20%, especially for more centrally located tumors, where its role is constrained due to the elevated risk of grade ≥ 3 pulmonary fibrosis and major pulmonary hemorrhage [6]. Its toxicity may be fatal in patients with pre-existing interstitial lung fibrosis [6]. Moreover, it detrimentally affects pulmonary function, reducing DLCO by up to 12%, which is particularly detrimental for patients with minimal respiratory reserves [4].

In this context, thermal ablation techniques are gaining more prominence as more data is published and they are included in all the relevant international guidelines as a treatment option for inoperable early-stage NSCLC [2,6]. However, those techniques require specialized centers and educated interventional radiologists and units for the hospitalization of the patients, which limit their application.

Herein, we present 2 cases of early-stage NSCLC with subsequent poor lung function that were successfully treated with CT-guided cryoablation.

## Case report 1

### Patient presentation, clinical findings and diagnostic assessment

The first case involves a 71-year-old Caucasian man, an active smoker with a history of 70 pack-years (py). His medical history includes chronic obstructive pulmonary disease (COPD), requiring long-term oxygen therapy, obstructive sleep apnea (OSA) managed with continuous positive airway pressure (CPAP), arterial hypertension, a surgically treated thoracic aortic aneurysm 1 year ago and severe heart failure (HF), confirmed by a recent cardiological checkup.

**Fig. 1:**
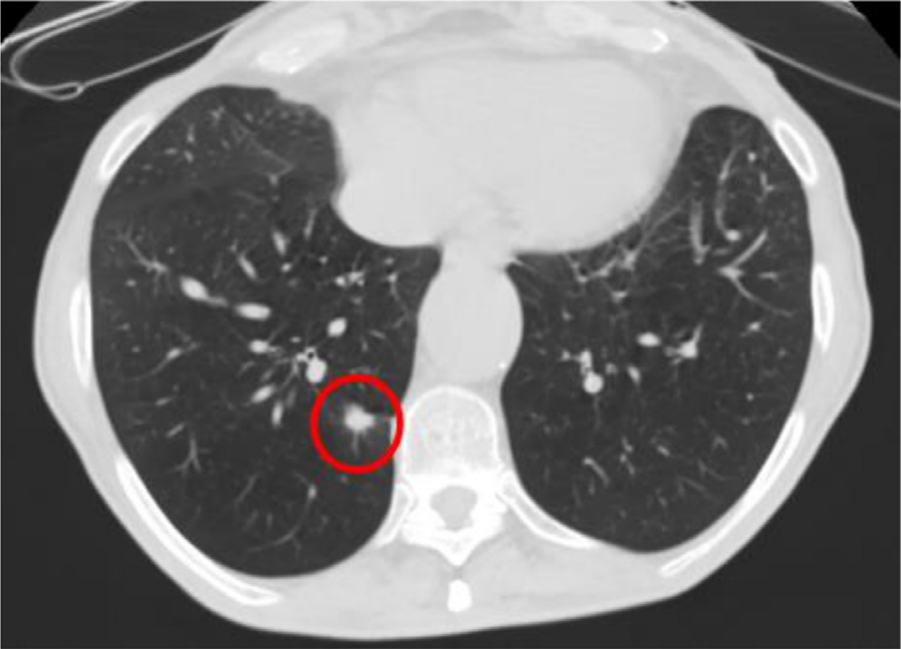
Thoracic CT scan of the first patient, with the pulmonary lesion of the right lower lobe highlighted within the red circle. The lesion measures 1.3 × 1.5 cm.

After repeated episodes of hemoptysis, the patient underwent a CT scan of the thorax, which revealed a 1.3 × 1.5 cm pulmonary mass with spiculated margins in the right lower lobe (Fig. 1). Taking into account the smoking history of the patients and the characteristics of the lesion, the Brock model estimated a 25% probability of malignancy, and further evaluation was suggested. The PET scan and the magnetic resonance imaging (MRI) of the brain were negative for distal or lymph node metastases. Due to the fact that the tumor was less than 3 cm with peripheral distribution and without lymph node involvement on imaging, no further mediastinal staging was needed.

The patient did not wish to proceed with any therapeutic intervention before confirming the nature of the lesion and therefore underwent a CT-guided biopsy, which was indicative of lung adenocarcinoma. The molecular and biomarker analyses of the tumor were negative. According to the eighth edition of the TNM staging for lung cancer, the patient had stage IA lung adenocarcinoma (T1bN0M0) [7]. A recent spirometry test revealed severe obstructive disorder in the pulmonary function (FEV1:600 mL-19% of normal, Forced Vital Capacity (FVC):1.99 mL-48% of normal, FEV1/FVC:30.2% of normal, DLCO:28% of normal). This, combined with all the serious underlying health conditions, made the patient ineligible for surgery [8].

### Therapeutic intervention and follow up

The multidisciplinary team (MDT) discussion recommended definitive local treatment, prioritizing IGTA, as it only temporarily affects spirometric measures (FEV1, DLCO) and can be performed at our medical center by a specialized team of interventional radiologists.

**Fig. 2:**
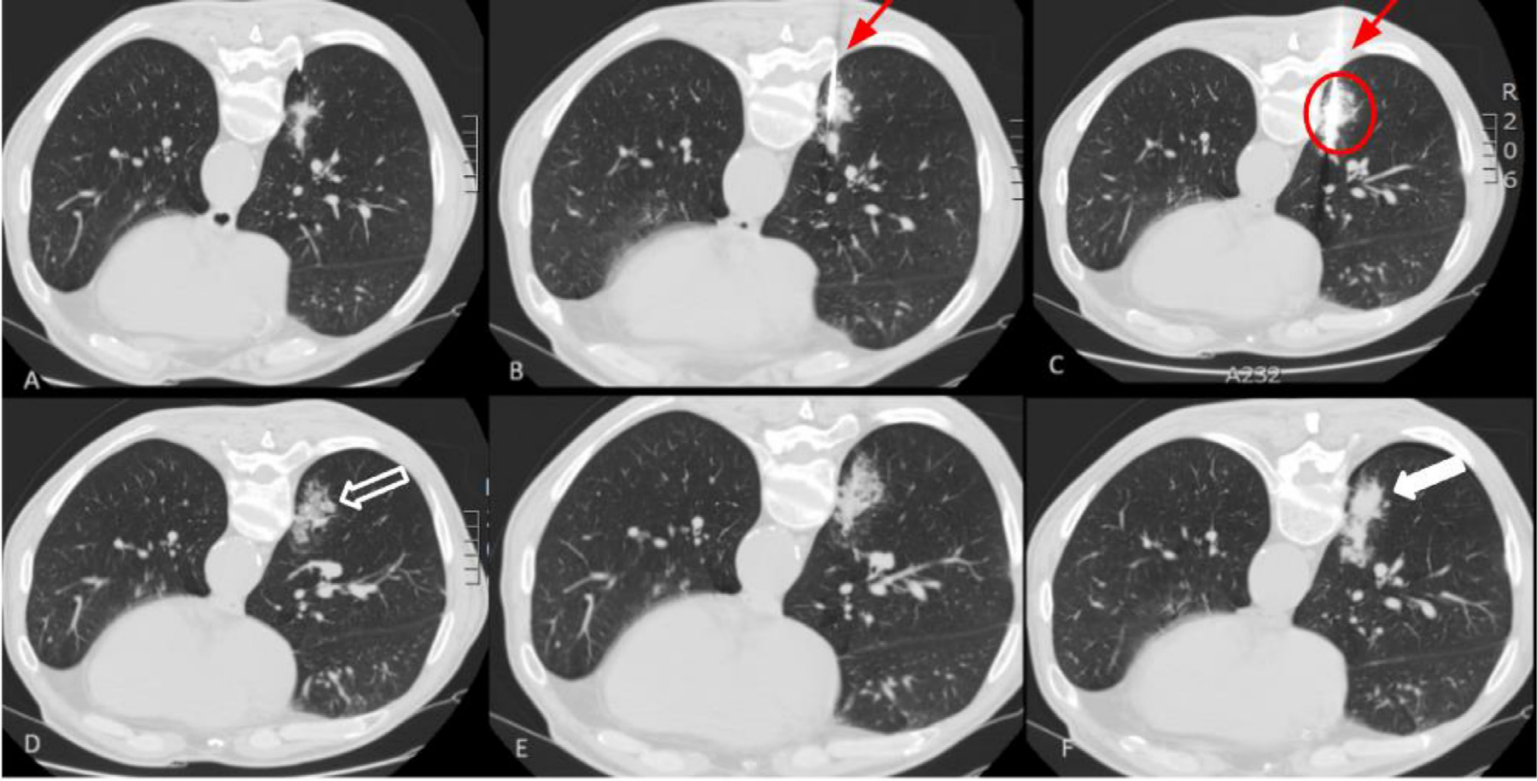
Images of the first patient during the cryoablation. The CT images are presented in interventional orientation, where posterior anatomical structures appear at the top of the image. (A-C) The images depict the third and final cycle of the freezing-thawing process, illustrating the formation of the ice ball, representing the ablation zone (area into the circle). This clearly demonstrates the minimal extent of parenchymal invasion associated with the procedure. The insertion of the needle into the mass is visible (red arrow). (D-F) This results in a region of ground-glass opacity (empty arrow) and consolidation (white arrow) around the ablation (CT scan immediately following the procedure) as the melting ice induces coagulation necrosis, alveolar hemorrhage, and edema.

The patient underwent cryoablation (Fig. 2). A small pneumothorax occurred postintervention that was treated conservatively and the patient was hospitalized for a day for monitoring purposes.

**Fig. 3:**
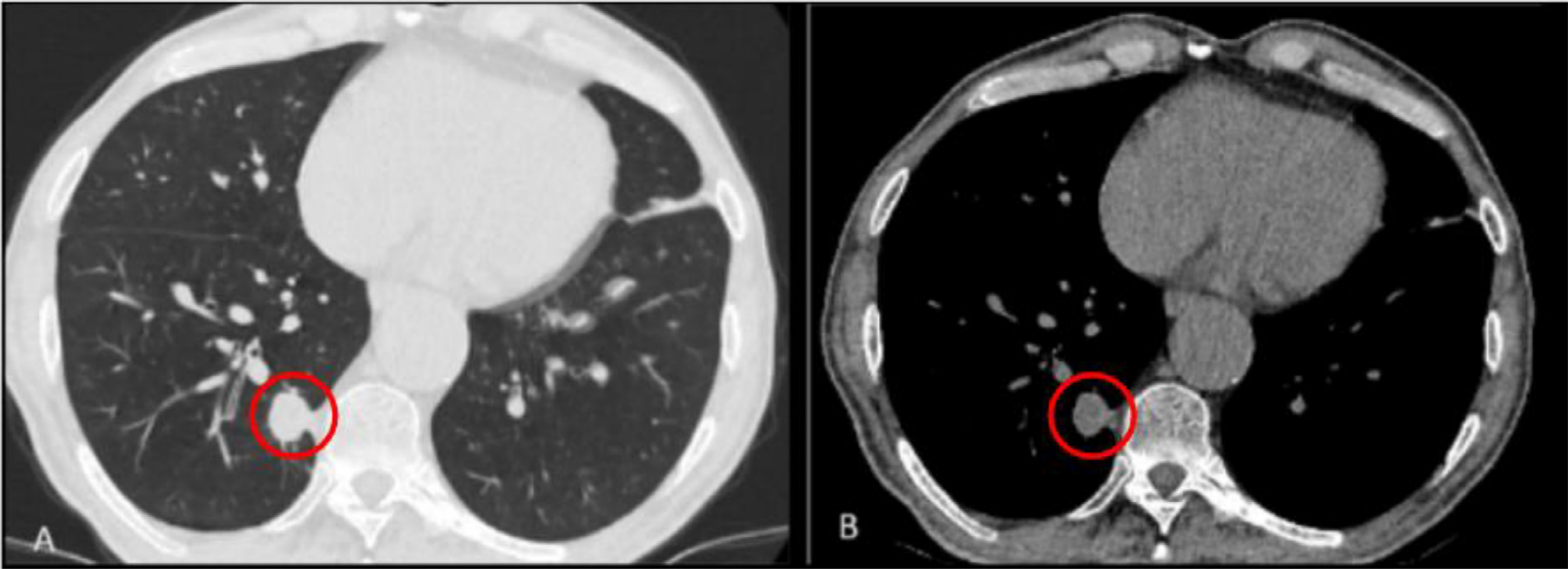
(A) Lung window of the thoracic CT of the first patient 1 month after the cryoablation B) Mediastinal (soft tissue) window of the same thoracic CT. Normally, an early increase in surface area is seen, which peaks at 1 month. During the healing process, the ablation zone is transitioning from a ground-glass appearance to a more defined, nodular pattern (area into the circle).

The follow-up thoracic CT scan in 1 and 3 months showed no local recurrence nor residual tumor (Fig. 3). Six months postprocedure, a PET scan was conducted that showed no metabolic uptake of the primary tumor in the right lower lobe; however, it revealed a lytic lesion on the fifth thoracic vertebra (T5), indicative of potential metastatic disease. Even though the patient did not present any related symptoms, regional radiotherapy combined with radiofrequency ablation (RFA) and vertebroplasty for stabilization were performed in order to avoid pathological fractures and achieve local tumor control.

**Fig. 4:**
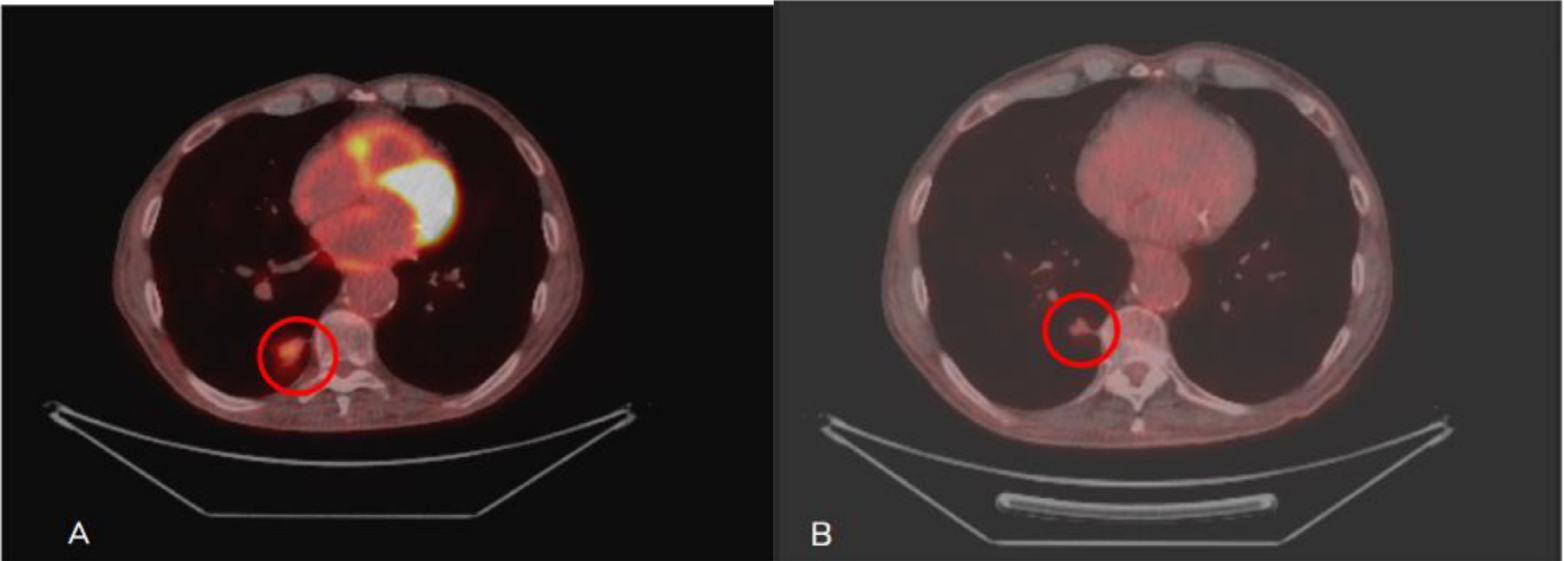
(A) PET/CT scan of the first patient at the time of diagnosis, revealing the lesion in the right lower lobe, with poor metabolic uptake (area into the circle). (B) PET/CT scan 6 months postcryoablation, showing no metabolic uptake at the primary tumor site (area into the circle).

The patient's performance status and quality of life remained consistent with those at the time of diagnosis. These factors were primarily influenced by his extensive medical history and did not worsen following treatment. As a result, we decided to continue with regular follow-up. On the follow-up, 1 year after the cryoablation, the PET scan revealed no residual metabolic activity of the primary tumor nor of the metastasis (Fig. 4).

**Fig. 5:**
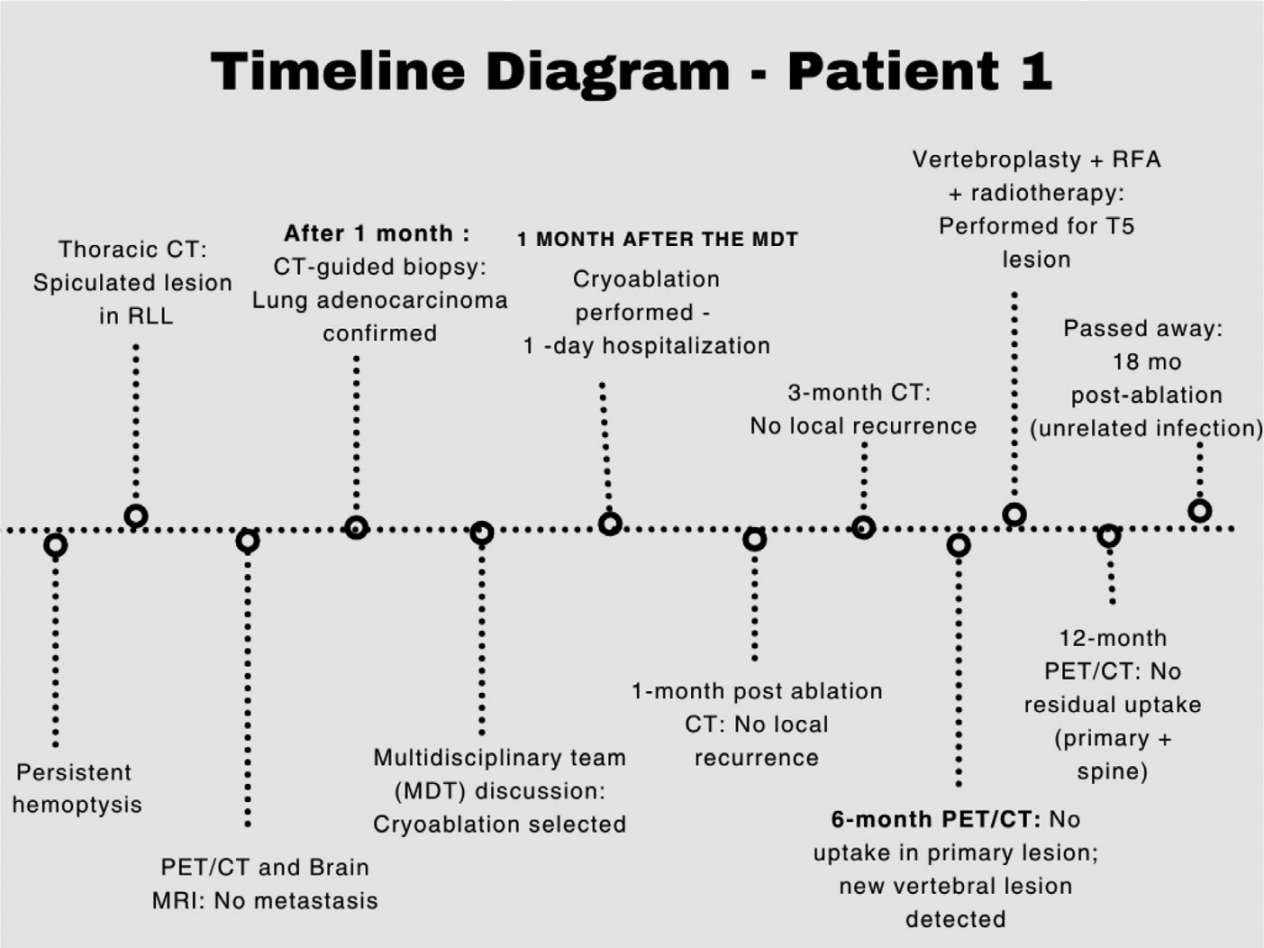
Timeline diagram of the first patient’s diagnostic process, treatment, and follow-up. Actual dates are not shown; the diagram illustrates the intervals between key events and imaging follow-up.

Unfortunately, he passed away 6 months later due to a lower respiratory tract infection, before his scheduled follow-up CT scan. The patient’s diagnostic and treatment timeline is shown in Fig. 5.

## Case report 2

### Patient presentation, clinical findings and diagnostic assessment

The second case involves a 78-year-old man with a heavy smoking history of 120 py without other known comorbidities. The patient presented to the outpatient clinic with a confirmed diagnosis of lung adenocarcinoma, established through examinations previously arranged by the attending internal medicine physician due to a persistent cough. More specifically, a thoracic CT scan revealed a 16-mm lesion in the upper lobe of the left lung and the PET scan that followed showed no pathological uptake except for the previously identified lung lesion. The MRI of the brain showed no evidence of metastasis. Based on these findings, the patient was diagnosed with stage IA2 (T1bN0M0) lung adenocarcinoma [6].

Similar to the first patient, he also exhibited a severe obstructive pattern on spirometry (FEV1: 440 ml (15%), FVC: 2130 ml (48%), FEV1/FVC: 20.7%). Unfortunately, the patient could not perform the DCLO test due to respiratory muscle fatigue. Additionally, the performance status of the patient according to the WHO/ECOG scale was 2, indicating a symptomatic patient who spent <50% of the day in bed [7].

### Therapeutic intervention and follow-up

**Fig. 6:**
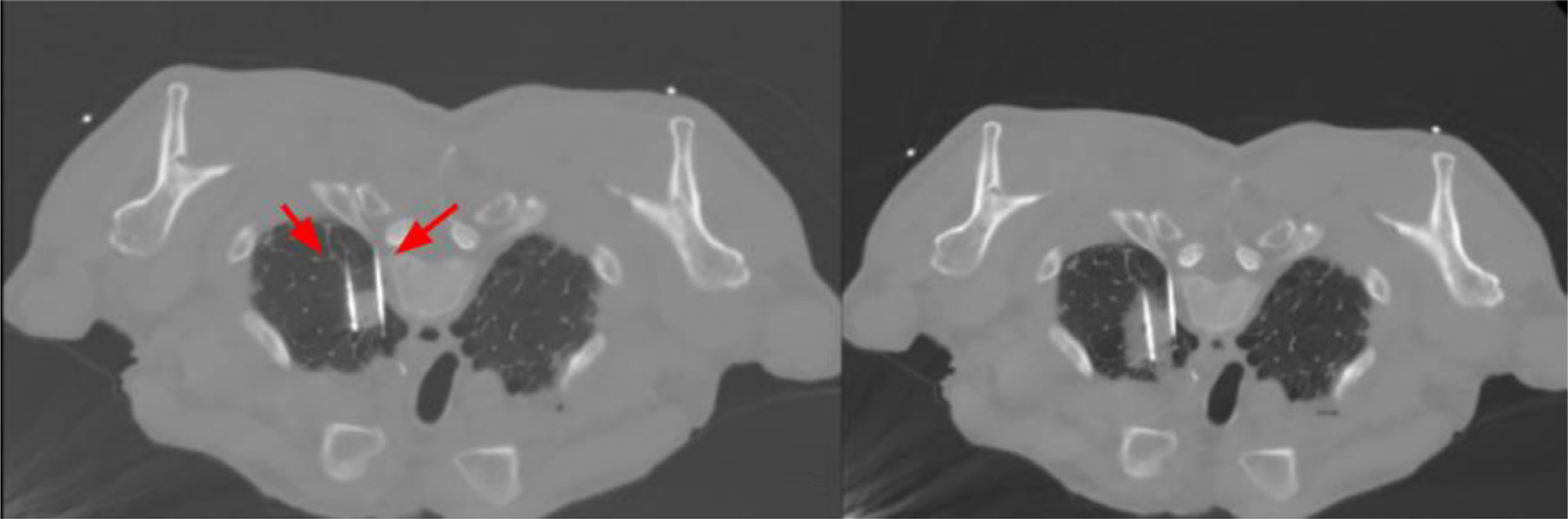
Thoracic CT of the second patient during the cryoablation (mediastinal window). In this patient, 2 needles (red arrows) are used in order to create an ablation zone with safety margins due to its size. The CT images are presented in interventional orientation, where posterior anatomical structures appear at the top of the image.

Taking into account his advanced age, impaired lung function, overall clinical status, and the MDT’s recommendation—based on similar considerations as in the first patient—thermal ablation was selected as the most appropriate treatment option (Fig. 6). He did not experience any complications postprocedure and was hospitalized for 1 day just for monitoring.

**Fig. 7:**
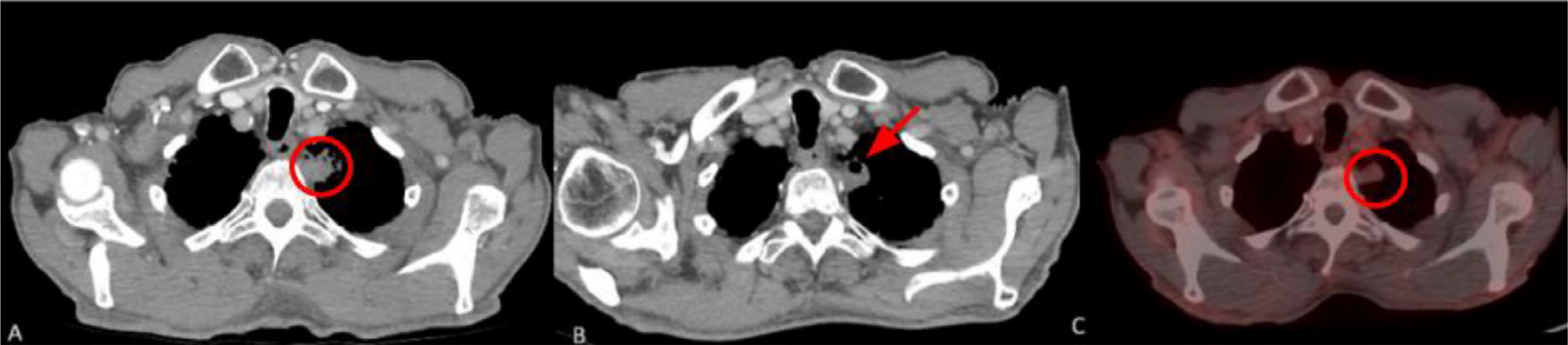
Follow-up images from the second patient. (A) 1-month postablation thoracic CT scan showing no enhancement of the ablated lesion consistent with absence of residual tumor or local recurrence. The area demonstrates a nodular pattern (area into the circle). (B) The thoracic CT 3 months after the cryoablation shows a small cavitation in the ablated nodule (red arrow), which is common as it appears in about half of the patients and is typically resolved within 6 months. (C) The PET/CT scan 6 months postcryoablation revealed no Fluorodeoxyglucose (FDG) uptake of the lesion (area into the circle), as well as no evidence of distant metastases .

**Fig. 8:**
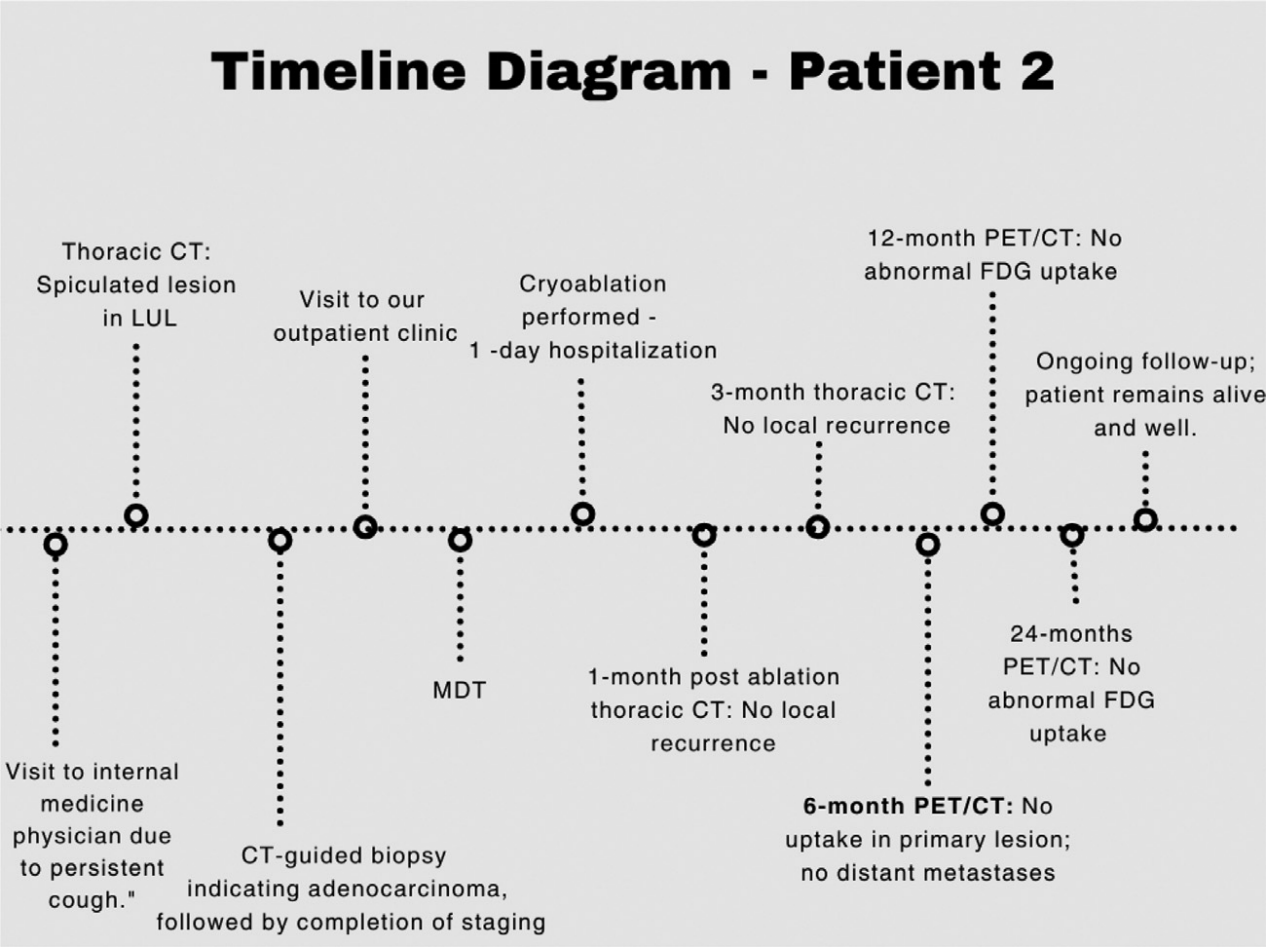
Timeline of the second patient’s diagnostic evaluation, treatment, and follow-up. The patient remains alive and under regular clinical follow-up.

The follow-up CT scan that was conducted 1 and 3 months after the procedure revealed no residual tumor and no signs of local recurrence. Six months postcryoablation, the PET scan revealed no metabolic uptake at the lesion site or any other areas of the body (Fig. 7). As of today, 2 years after the procedure, the patient remains clinically stable, without any sign of local or distant metastases. The timeline diagram of the patient’s diagnostic process, treatment, and follow-up is displayed in Fig. 8.

## Discussion

As previously noted, the increasing use of lung cancer screening has led to a higher diagnosis rate of early-stage lung cancer. In cases where surgical intervention is not feasible, IGTA techniques are employed and are available at specialized centers globally. Given the high mortality and recurrence rates associated with lung cancer, it is imperative for all relevant medical specialties to be well-informed about the available treatment options. This ensures that patients are not excluded from potentially life-saving therapies, even if they have extensive medical histories.

There are 3 different types of IGTA: radiofrequency ablation (RFA), microwave ablation (MWA), and cryoablation, which share a common mechanism of utilizing extreme temperatures to induce tissue damage [5]. Among these, RFA is the most studied and utilized, with a predominant presence in the literature, as the first 3 cases were published in 2000 by Dupuy et al. [9,10]. Results from a prospective multi-center trial involving 54 patients with medically inoperable stage I NSCLC who underwent RFA highlighted overall survival of approximately 90% at 1 year, 70% at 2 years and 60% at 3 years [11]. There are currently no publications on MWA for primary lung cancer that involve patient follow-up periods exceeding 3 years.

Hence, for inoperable early-stage NSCLC, the ESMO guidelines recommend RFA as an alternative to SBRT. Conversely, NCCN guidelines consider IGTA an equal option when the necessary conditions are fulfilled and refrain from favoring a specific technique, emphasizing the importance of factors such as the size and location of the target lesion, the risk of complications, and the experience and familiarity of the chosen therapeutic center in determining the appropriate method [2,6].

When applied to treat lung cancer, these techniques refer to patients with primary stage I NSCLC, ideally < 3 cm , or metastatic lung tumors [2,12]. There have been published efforts addressing bigger lesions; however, studies have shown that smaller tumor sizes are associated with better therapeutic outcomes [11]. Overall, the procedure demonstrates excellent tolerance, with the only absolute contraindication being the presence of untreatable coagulopathies [12]. They do not adversely impact pulmonary function, but instead there are studies highlighting an improvement in FVC so they are extremely useful for cases where the respiratory reserves are significantly reduced [12,13]. The prognosis appears comparable to stereotactic radiation therapy, as demonstrated by 2 similar National Cancer Database studies, which conclude that the overall survival rates are similar, even though patients who underwent ablation had more comorbidities [14,15].

Our hospital has great expertise in cryoablation, so this was the selected technique. It consists of a triple freeze protocol with 3 cycles of freezing and thawing. At temperatures as low as -160°C, ice crystals are formed, leading to membrane rupture, osmotic shock, and vascular endothelial cell damage with platelet stasis, microthrombi formation, and consequent ischemia and cell death [5,12]. It is delivered percutaneously under image guidance. Cryoablation can be safely performed under local anesthesia alone, and this is another reason why it is preferred. Also, it can address larger masses by using more than 1 cryoprobe, as performed on 1 of our patients, has the ability to treat central located tumors and has a very low complication rate compared to other thermal ablative modalities. One of its greatest advantages is the ability to visualize the ablation zone as a ground glass opacity on the CT immediately after ablation which is helpful to achieve A0 margins (Fig. 4) [12].

The most common side effect is pneumothorax; however, less than 20% of the cases require intervention with chest tube placement. When significant, pneumothorax can be promptly managed by the interventional radiologists through immediate aspiration or in situ chest tube placement, allowing for rapid resolution of the complication. 2% will develop major complications such as pulmonary hemorrhage requiring transfusion, embolization, or bronchoscopic management, diaphragmatic and nerve injuries, or later onset pneumonia, abscess, and bronchopleural fistula. Usually only 1 hospital day is required for follow-up, while mortality is extremely rare [9,12].

Based on the published literature, the outcomes and effectiveness of cryoablation are equally encouraging. A retrospective study by Moore et al. showed a 5-year survival rate of 67.8% after cryoablation in medically inoperable stage I NSCLC, with 85% of the patients showing no evidence of recurrence [16]. This survival rate is twice that of SBRT and comparable to surgery, as lobectomy features a 5-year survival rate of 60-80% while sublobar resection has a slightly more modest 40-60%. In addition, a recent meta-analysis concluded that cryoablation was superior to RFA in terms of 3-year disease free-survival and complication rates [17].

Upon completion of the procedure, a chest x-ray within 4 hours must be performed in order to exclude the presence of pneumothorax [12]. In terms of the follow-up, there is no standard imaging protocol; however, there are some general principles adjusted to each center’s approach. So usually, a contrast-enhanced CT scan should be performed at 1, 3, 6, 9, 12 months, and then yearly, and an FDG-PET/CT scan at 6 and 12 months [12,16].

After ablation, specific imaging patterns emerge, irrespective of whether it is hot-based or cold-based. In the case of cryoablation, immediately following the procedure, a zone of ground glass opacity and consolidation will manifest around the ablation site as the melting ice induces coagulation necrosis, alveolar hemorrhage, and edema [9]. This pattern was clearly noted in both of our patients CT scans at the end of the process (Fig. 2). The early increase in surface area usually peaks at 1 month and is subsequently followed by a gradual decrease, although the ablation zone may still be larger than the original tumor [18]. In about half of the patients, cavitation will appear within the region of the ablated nodule but is typically resolved within 6 months. In noncontrast CT follow up, a decreased nodule density is desirable because it is associated with complete ablation of the tumor [18].

In the course of healing, the ablation zone undergoes some shape transformations and is classified into 1 of 5 patterns: consolidation/atelectasis, nodular pattern, stripe pattern, pleural thickening, and disappearance [19]. Consolidation/atelectasis and the nodular pattern are prevalent, while pleural thickening and disappearance indicate complete local control. The stripe pattern poses challenges as it is considered a scar against typical tendencies, so strict follow-up is essential [18,19].

Τhermal ablation provides the privilege of repeatability of treatment in cases of local reccurrence, given the absence of the plateau effect seen in radiotherapy [9,11]. In addition to this, even in recurrences needing systemic therapy, these are minimally invasive, well tolerated methods that do not alter the patient's already aggravated condition [11]. Its flexibility in adapting to different tumor sizes and locations is also noteworthy; for example, in our second case, we successfully used 2 cryoprobes to treat a larger lesion, demonstrating how the technique can be tailored to individual patient needs.

Considering that thermal ablation creates an immunologic cascade with an influx of inflammatory and antigen-presenting cells, and the subsequent death results in the release of a variety of cytokines and other signs that alter the immune system and initiate a cellular and/or humoral immune response, there has been recent scientific interest in the possible synergistic action of ablation techniques with immunotherapy [[5], [20], [21], [22]]. Feng et al. compared retrospectively the combination of cryoablation and nivolumab versus nivolumab alone in patients with advanced NSCLC and concluded that the combination therapy led to a notable reduction in circulating tumor cells and lowered the level of tumor markers without statistically differentiating the presence of adverse events [21]. There are numerous unanswered questions about the indications and applications of this combination; however, these data emphasize that there is scope for further research [21].

The main limitation of this manuscript is that it presents only 2 cases. However, these 2 cases underscore the expanding role of cryoablation as a safe and effective local treatment option for early-stage NSCLC in patients who are not eligible for surgery. Despite significant comorbidities and severely impaired respiratory function, both patients tolerated the procedure well, with no further decline in quality of life. This population often includes relatively young individuals whose daily functioning is already compromised due to underlying cardiopulmonary conditions. In such cases, it is essential to offer treatment strategies that are both effective and minimally invasive, avoiding further deterioration of their overall condition.

## Author contributions

All authors contributed equally to this work. Specifically, E. Eleftheriadou and D. Samara were responsible for data collection and the writing process. M. Saroglou conceptualized and designed the article, as well as made the final revisions. E. Petsatodis provided all images and reviewed and revised the manuscript. All authors have approved the final version for publication, agreed on the journal to which the article has been submitted, and are accountable for all aspects of the work.

## Patient consent

Informed consent for the publication of this case report was obtained from the patients. They were made aware of the nature of the publication, including the use of their medical information, and were informed of their right to confidentiality. We confirm that all necessary precautions have been taken to ensure the protection of their privacy, and their identities will remain confidential in line with relevant ethical guidelines and regulations. The signed consents are available upon request.
